# Physical and Flavor Characteristics, Fatty Acid Profile, Antioxidant Status and Nrf2-Dependent Antioxidant Enzyme Gene Expression Changes in Young Grass Carp (*Ctenopharyngodon idella*) Fillets Fed Dietary Valine

**DOI:** 10.1371/journal.pone.0169270

**Published:** 2017-01-24

**Authors:** Jian-Bo Luo, Lin Feng, Wei-Dan Jiang, Yang Liu, Pei Wu, Jun Jiang, Sheng-Yao Kuang, Ling Tang, Wu-Neng Tang, Yong-An Zhang, Xiao-Qiu Zhou

**Affiliations:** 1 Animal Nutrition Institute, Sichuan Agricultural University, Chengdu, China; 2 Fish Nutrition and Safety Production University Key Laboratory of Sichuan Province, Sichuan Agricultural University, Chengdu, China; 3 Key Laboratory for Animal Disease-Resistance Nutrition of China Ministry of Education, Sichuan Agricultural University, Chengdu, China; 4 Animal Nutrition Institute, Sichuan Academy of Animal Science, Chengdu, China; 5 Institute of Hydrobiology, Chinese Academy of Sciences, Wuhan, China; Universidade de Vigo, SPAIN

## Abstract

This study was conducted to examine the effects of dietary valine on the physical and flavor characteristics, fatty acid (FA) profile, antioxidant status and Nrf2-dependent antioxidant enzyme gene expression in the muscle of young grass carp (*Ctenopharyngodon idella*) fed increasing levels of valine (4.3, 8.0, 10.6, 13.1, 16.9 and 19.1 g/kg) for 8 weeks. Compared with the control group, the group fed valine showed improved physical characteristics of fish fillets (increased relative shear force, hydroxyproline, protein and lipid levels and decreased cathepsin B and L activities, as well as cooking loss, were observed). Moreover, valine improved the flavor of young grass carp fillets by increasing the amino acid (AA) concentration in fish muscle (increased aspartic acid, threonine, glutamine, cystine, methionine, leucine, tyrosine, phenylalanine, lysine, histidine, arginine and valine concentrations were observed). Additionally, optimal valine supplementation increased the potential health benefits to humans by decreasing the saturated FA (C15:0 and C16:0) concentration and increasing the unsaturated FA (monounsaturated FAs (MUFAs), such as C16:1, C18:1c+t and C20:1, and polyunsaturated FAs (PUFAs), such as C18:3n-3, C20:2 and C22:6) concentration. In addition, the reduced glutathione (GSH) content and the activities of Cu/Zn superoxide dismutase (SOD1), catalase (CAT) and Selenium-dependent glutathione peroxydase (Se-GPx) increased under valine supplementation (*P* < 0.05). Furthermore, the SOD1, CAT and Se-GPx mRNA levels increased with dietary valine levels, possibly due to the up-regulation of NF-E2-related factor 2 (Nrf2), target of rapamycin (TOR) and ribosomal protein S6 kinase 1 (S6K1) and the down-regulation of Kelch-like-ECH-associated protein 1 (Keap1) in muscle (*P* < 0.05). In conclusion, valine improved the physical and flavor characteristics, FA profile, and antioxidant status and regulated the expression of the antioxidant enzyme genes Nrf2, Keap1, TOR and S6K1 in fish fillets.

## 1. Introduction

In China, farmed fish account for more than 70% of the total food fish supply [1]. Grass carp is a native Chinese freshwater fish that is widely cultured in China because of its good taste and high nutritional value [2]. However, the deterioration of flesh quality is one of the most important issues facing the aquaculture industry, as it leads to sensory, physical and nutritional quality losses and generates off-flavors, thereby causing tremendous economic losses [3]. Hence, improved methods are needed to protect fish muscle from flesh quality deterioration to improve meat quality.

Nutrient supplementation has been suggested as safe ways to improve fish flesh quality and to retard deterioration [4]. Valine has been discovered to be an essential amino acid for fish growth and primarily deposited in body protein, notably in skeletal muscle protein [5]. The relative shear force, water-holding capacity (WHC) and pH are the most important physical characteristics of fish flesh [6] because the firmness, water content and post-mortem pH in fish muscle are positively correlated with good mouthfeel [7]. Antioxidative defenses in fish are directly proportional to the relative shear force and WHC [8]. It has been reported that valine is a major substrate for the synthesis of α-melanocyte-stimulating hormone (α-MSH) in fish [9]. In both keratinocytes and melanocytes, α-MSH significantly improves antioxidant status and increases γ-glutamylcysteine-synthetase and glutathione (GSH)-S- transferase Pi gene expression [10]. In addition, valine inhibits deoxyribose attack by •OH, which is induced by Fenton-like reactions *in vitro* [11]. We previously found that valine improves the growth and development of the intestines [12] and gills [13] of grass carp. The growth and development of these organs depend on the structural integrity of cells and have been associated with antioxidant defenses [14]. Based on these studies, we speculate that dietary valine may influence flesh quality parameters and warrants further investigation. In addition to the mouthfeel of fish, flavor is a key flesh quality characteristic that confers a high market value and consumer demand [15]. Free amino acids (AAs), such as glutamate, aspartic acid, glycine, alanine and histidine, contribute directly to flavor development [16, 17]. Harper et al. [18] suggested that a better amino acid (AA) balance could improve AA accumulation. A better AA balance in the diet reduces the plasma ammonia content (PAC) in fish [19]. We previously found that valine decreases the PAC in grass carp [12]. In addition, the absorption of AAs requires an extracellular sodium ion concentration gradient [20] and is positively correlated with Na^+^, K^+^-ATPase activity [21]. In juvenile Jian carp (*Cyprinus carpio var*. Jian), an optimum valine diet increases the Na^+^, K^+^-ATPase activity [22]. These data suggest that valine may affect the AA content in fish, but no studies have investigated this hypothesis. Moreover, the edible parts of fish are of particular interest for human consumption due to their richness in polyunsaturated fatty acids (PUFAs), such as eicosapentaenoic acid (EPA; C20:5) and docosahexaenoic acid (DHA; C22:6) [23]. FA synthetase (FAS) is a critical enzyme for lipogenesis [24] and plays a key role in catalyzing the synthesis of PUFAs [25]. In mouse liver, valine deprivation suppresses FAS expression [26]. These data indicate that valine might affect FA content and requires further investigation.

As mentioned above, physical characteristics in fish fillets are in relation with antioxidant defenses. In general, fish antioxidant systems are composed of non-enzymatic compounds (glutathione, GSH) and antioxidant enzyme (superoxide dismutase, SOD; catalase, CAT; Selenium-dependent glutathione peroxydase, Se-GPx) activities [27]. In mice, valine increases the capacity for ROS elimination in the heart and soleus muscles [28]. In fish, ROS scavenging ability is strongly correlated with antioxidant defenses [29], which suggests that dietary valine may have beneficial effects on fish antioxidant defenses and requires further investigated. Furthermore, Nrf2 (NF-E2-related factor 2)-Keap1 (Kelch-like ECH-associated protein 1) signaling plays a crucial role in the coordinated induction of antioxidant enzymes, such as Cu/Zn SOD1, CAT and Se-GPx in fish [30]. To date, the effects of valine on the Nrf2 signaling pathway in fish muscle are unknown. The mammalian target of rapamycin (mTOR) and its major downstream target protein ribosomal protein S6 kinase 1 (S6K1) [31] are key regulators of Nrf2 expression [32]. In mice, branched-chain AA (BCAA) supplementation increases the activation of mTOR and S6K1, the best-characterized mTOR substrate [28]. Thus, valine may affect Nrf2 expression by modulating the TOR signaling pathway; however, this relationship has not been investigated.

This study was a part of a larger study investigating the effects of valine on the development of the intestines [12] and gills [13] of fish and uses the same growth trial used in a previous study. In this study, we investigated the effects of valine on the physical (pH, relative shear force, cooking loss) and flavor (AA) characteristics, FA profile, antioxidant status and Nrf2-dependent antioxidant enzyme gene expression in the muscle of grass carp; this information could be used to preliminarily identify a valine-dependent mechanism that improves fillet quality.

## 2. Materials and methods

### 2.1. Ethics statement

All experimental protocols were approved by the Animal Care Advisory Committee of Sichuan Agricultural University.

### 2.2. Experimental design and diets

The composition of the basal diet is the same as that used in our previous study [12] and is presented in Table 1. In brief, fish oils and soybean oil were used as dietary lipid sources. Fishmeal, casein and gelatin were used as the main protein sources. Crystalline AAs were used to adjust the AA profile of the diets to 30% whole chicken egg protein, excluding valine, as described by Abidi et al. [33]. In our previous study, optimal fish growth occurred with a diet of 14.00 g/kg valine [12]. Therefore, in this study, fish were fed six different diets that were developed by supplementing the basal diet with valine at concentrations of 0 (un-supplemented control), 3, 6, 9, 12, and 15 g/kg valine. The diets were made isonitrogenous through supplementation with glycine, as described by Chen et al. [34]. The pH of each diet was adjusted to 7.0 by gradually adding 6.0 M NaOH according to Dong et al. [22]. The feed was produced and stored at -20°C, as described by Shiau et al. [35]. The valine concentrations in the experimental diets were measured to be 4.3, 8.0, 10.6, 13.1, 16.7 and 19.1 g/kg, as described by Ahmed et al. [36].

**Table 1:** Composition and nutrients content of the basal diet.

Ingredient	g kg^-1^	Nutrients content ^a^	g kg^-1^
Fish meal	78.00	Crude protein	293.8
Casein	30.00	Crude lipid	47.6
Gelatin	39.90	Valine	4.3
Amino acid mix^b^	152.10	n-3	10
Valine premix^c^	50.00	n-6	10
Glycine premix^d^	70.00	Available phosphorus	6
Corn starch	99.90		
α-starch	280.00		
Fish oil	22.00		
Soybean oil	18.90		
Vitamin premix^e^	10.00		
Trace mineral premix^f^	20.00		
Ca(H_2_PO_4_)_2_	22.70		
Choline chloride (50%)	6.00		
cellulose	100.00		
Ethoxyquin (30%)	0.50		

^a^Crude protein and crude lipid were measured value. Available phosphorus, n-3 and n-6 contents were calculated according to NRC (1993).

^b^Amino acid mix (g kg^-1^ diet): lysine, 17.13; methionine, 7.78; tryptophan, 3.57; threonine, 11.88; arginine, 12.89; histidine, 7.96; leucine, 20.51; isoleucine, 12.69; phenylalanine, 13.60; tyrosine, 10.86; glutamic acid, 32.32; Cysteine, 0.91, respectively.

^c^L-valine was added to obtain graded levels of valine. Per kilogram of valine premix composition from diet 1 to 6 was as follows (g kg^-1^): L-valine (98.5%) 0.00, 60.91, 121.83, 182.74, 243.65, 304.57 g and corn starch 1000.00, 939.09, 878.17, 817.26, 756.35, 695.43 g, respectively.

^d^Each mixture was made isonitrogenous with the addition of reduced amounts of glycine and compensated with appropriate amounts of corn starch. Per kilogram of glycine premix composition from diet 1 to 6 was as follows (g kg^-1^): glycine (99%) 859.83, 832.09, 804.35, 776.61, 748.87, 721.13 g and corn starch 140.17, 167.91, 195.65, 223.39, 251.13, 278.87 g, respectively.

^e^Per kilogram of vitamin premix (g kg^-1^): retinyl acetate (500000IU g^-1^), 0.800 g; cholecalciferol (500000IU g^-1^), 0.480 g; D,L-a-tocopherol acetate (50%), 20.000 g; menadione (23%), 0.220 g; thiamine hydrochloride (98%), 0.120 g; riboflavin (80%), 0.990 g; pyridoxine hydrochloride (98%), 0.620 g; cyanocobalamin (1%), 0.100 g; niacin (99%), 2.58 g; D-biotin (2%), 5.000 g; meso-inositol (99%), 52.330 g; folic acid (96%), 0.520 g; ascorhyl acetate (93%), 7.160 g; calcium-D-pantothenate (90%), 2.780 g; corn starch 906.3 g.

^f^Per kilogram of mineral premix (g kg^-1^): FeSO_4_·H_2_O, 25.00 g; CuSO_4_·5H_2_O, 0.60 g; ZnSO_4_·H_2_O, 4.35 g; MnSO_4_·H_2_O, 2.04 g; KI, 1.10 g; NaSeO_3_, 2.50 g; MgSO_4_·H_2_O, 230.67 g; corn starch 733.74 g.

### 2.3. Feeding management

The procedures used in this study were conducted in accordance with the University of Sichuan Agricultural Animal Care Advisory Committee. Grass carp were obtained from the Lu-ban Lake Fisheries (Sichuan, China). Fish were adapted to the experimental environment for 2 weeks. A total of 540 fish with an average initial weight of 268 ± 2.25 g were randomly assigned to 18 experimental cages (1.4 × 1.4 × 1.4 m^3^), each of which was equipped on the bottom with a disc of 80 cm in diameter and randomly assigned to one of three replicates of the six dietary treatments, as described by Tang et al. [37]. The water temperature, pH and dissolved oxygen content were 25 ± 2°C, 7.5 ± 0.3 and 5.0 ± 0.5 mg L^-1^, respectively. The fish were fed the respective diets four times daily for 8 weeks. Thirty minutes after feeding, uneaten feed was collected, dried and weighed to calculate the feed intake, as described by Mundheim et al. [38].

### 2.4. Tissue sample collection

Fish from each cage were weighed at the beginning and at the end of the feeding trial. A total of fifteen fish collected from each treatment were anaesthetized in a benzocaine bath (50 mg/L) 12 h after the last feeding, as described by Berdikova Bohne et al. [39], and the fish were stunned by a sharp blow to the head with a wooden stick [40]. Immediately after death, the left side muscle of the first nine fish from each treatment was quickly removed and stored at -80°C in liquid nitrogen to analyze the fillet composition and antioxidant parameters. Another six fish from each treatment were randomly collected and tested for fillet quality. The weight of the fish sampled fed diets supplemented with 4.3, 8.0, 10.6, 13.1, 16.7 and 19.1 g/kg valine were shown in Table 2 and S1 Table.

**Table 2:** The mean body weight (MBW, g fish^-1^) of the fish sampled.

Dietary valine levels (g kg ^-1^ diet)						
	4.3	8.0	10.6	13.1	16.7	19.1
MBW	360.2±5.8^a^	497.9±14.9^b^	613.9±24.8^e^	679.8±17.3^f^	593.2±3.6^d^	578.8±1.6^c^

Values are means ± SD (n = 15), and different superscripts (a,b,c) in the same row are significantly different (*P* < 0.05).

### 2.5. Analysis and measurement

The moisture content was estimated by oven drying the sample to a constant weight. The crude protein (N-Kjeldahl × 6.25) content was assayed using the Kjeldahl method after acid digestion. The crude fat content was determined using the Soxhlet exhaustive extraction technique with petroleum ether as the solvent. The hydroxyproline content of the muscle was determined according to the method of Periago et al. [41]. Six fish from each treatment were placed in polyethylene (PE) bags for qualitative evaluation. Twenty-four hours after slaughtering, each grass carp was stunned, deblooded, filleted and skinned. Then, the muscle pH was measured (WTW pH meter 197 with SenTix Sp and TFK 150/E) in three parts of the dorsal fillet. At 48 h post mortem, samples from the dorsal sections of fillets from 6 fish of each of the six treatments were heat sealed in duplicate in PE bags and heated to 70°C for 20 min. After cooling, the cooking loss was calculated according to Brinker et al. [42]. Samples were then collected using a blade of a Warner-Bratzler knife, and the relative shear force (N^−1^) of the cooked flesh was determined, as described by Morzel et al. [43]. In addition, a sample of 5–10 g was collected to measure the cathepsin B and L activities, as described by Kirschke et al. [44].

The AA composition of the fillets was determined according to Mostert et al. [45]. The amount of protein-bound AAs in the tissues was determined after hydrolysis in 6 N HCl for 24 h at 110°C in sealed tubes after replacing oxygen with nitrogen. The obtained solution was filtered through a 25-Am membrane filter and analyzed for AAs. Whole-muscle AAs were analyzed using an HITACHI Model L-8800 high-speed AA analyzer. To analyze the FA and lipid classes, fillet lipids were extracted according to Folch et al. [46] using the chloroform-methanol (2:1). The FA composition of the fats was determined using a gas chromatograph (GC-2010PLUS, SHIMADZU) equipped with an autosampler (AOC-20i+s). The GC oven temperature program was as follows: the initial temperature was held at 140°C for 5 min, increased to 180°C at 8°C/min, increased to 210°C at 4°C/min, increased to 250°C at 20°C/min, and then maintained for 7 min. The total running time was 26.5 min. The amount of each FA and its isomers was expressed as area percentage of FA methyl esters (FAMEs).

Tissue samples from six fish of each treatment were homogenized in 10 volumes (w/v) of ice-cold physiological saline and centrifuged at 6,000 g, 4°C for 20 min, after which the supernatants were collected to analyze the antioxidant parameters [47]. The muscle malondialdehyde (MDA) and protein carbonyl residue (PC) contents were assayed according to Zhang et al. [47], while the protein concentration of the tissue samples was determined according to Bradford et al. [48]. The activity of SOD1 was assayed by measuring the decrease in the rate of cytochrome c reduction in a xanthine-xanthine oxidase superoxide generating system [8]. The Se-GPx activity was determined by quantifying the rate of H_2_O_2_-induced oxidation of reduced GSH to oxidized glutathione [47]. The CAT activity was assayed according to Aebi et al. [49].

The formation of 5-thio-2-nitrobenzoate (TNB) was followed spectrophotometrically at 412 nm. The amount of GSH in the extract was assayed as mg/g protein using commercial GSH as the standard. The results are expressed as mg/g protein [50].

### 2.6. Real-time PCR analysis

The procedures for RNA extraction, reverse transcription and quantitative real-time PCR were similar to those described by Wu et al. [51]. Total RNA was extracted from the muscle using an RNAiso Plus kit (Takara, Dalian, Liaoning, China). The RNA quantity and quality were assessed by 1% agarose gel electrophoresis and spectrophotometry at 260 and 280 nm. Subsequently, cDNA synthesis was performed at 37°C for 15 min and 85°C for 5 s with 2 μl of total RNA using the PrimeScript^TM^ RT Reagent kit (TaKaRa Biotechnology (Dalian) Co., Ltd.) according to the manufacturer’s instructions. Specific primers for the Nrf2 and Keap1 genes were designed according to sequences of grass carp cloned in our laboratory (Table 3), and primers for SOD1, CAT, Se-GPx and β-Actin were designed using published sequences of grass carp (Table 3). Real-time PCR was performed for SOD1, CAT, Se-GPx, TOR, S6K1, Nrf2 and Keap1 according to standard protocols. Amplification was performed using SYBR Green PCR reagents and an Opticon DNA Engine (Bio-Rad) with the PCR amplification conditions shown in Table 3. After amplification, a melting curve analysis was performed to verify the accuracy of each amplicon over a range of 50–95°C. The expression levels of the SOD1, CAT, Se-GPx, TOR, S6K1, Nrf2 and Keap1 genes were normalized to the expression levels of the housekeeping grass carp β-actin gene. Each assay had five replications. In addition, the cDNA concentration in each sample was determined according to gene-specific standard curves. The standard curves were generated from both the target and an endogenous control based on 10-fold serial dilutions. The concentration of the target gene was calculated based on the threshold cycle number (CT), and the CT for each sample was determined using the MJ Option Monitor Software (version 3.1; Bio-Rad, Hemel Hempstead, Herts, UK). All standard curves had correlation coefficients greater than 0.99, and the corresponding real-time PCR efficiency values ranged between 0.90 and 1.10, as determined according to Chen et al. [34].

**Table 3:** Real-time PCR primer sequences.

Gene	Sequences of primers	Annealing temperature (°C)	Accession number
Nrf2^a^			
Forward	5/- CTGGACGAGGAGACTGGA-3/	62.5	KF733814
Reverse	5^/^- ATCTGTGGTAGGTGGAAC-3^/^		
Keap1^a^			
Forward	5^/^- TTCCACGCCCTCCTCAA-3^/^	63	KF811013
Reverse	5^/^- TGTACCCTCCCGCTATG-3^/^		
TOR^a^			
Forward	5^/^-TCCCACTTTCCACCAACT-3^/^	61.4	JX854449
Reverse	5/- ACACCTCCACCTTCTCCA-3/		
S6K1^a^			
Forward	5^/^-TGGAGGAGGTAATGGACG-3^/^	54	EF373673
Reverse	5^/^-ACATAAAGCAGCCTGACG-3^/^		
SOD1^a^			
Forward	5^/^- CGCACTTCAACCCTTACA-3^/^	61.5	GU901214
Reverse	5^/^- ACTTTCCTCATTGCCTCC-3^/^		
Se-GPx^a^			
Forward	5^/^- GGGCTGGTTATTCTGGGC-3^/^	61.5	EU828796
Reverse	5^/^- AGGCGATGTCATTCCTGTTC-3^/^		
CAT^a^			
Forward	5^/^-GAAGTTCTACACCGATGAGG-3^/^	58.7	FJ560431
Reverse	5^/^-CCAGAAATCCCAAACCAT-3^/^		
β-Actin			
Forward	5^/^- GGCTGTGCTGTCCCTGTA-3^/^	61.4	M25013
Reverse	5^/^- GGGCATAACCCTCGTAGAT-3^/^		

^a^Nrf2, NF-E2-related factor 2; Keap1, Kelch-like- ECH-associated protein 1; TOR, target of rapamycin; S6K1, S6 Kinase 1; SOD1, copper/zinc superoxide dismutase; CAT, catalase; Se-GPx, Selenium-dependent glutathione peroxydase

### 2.7. Statistical analysis

All results were expressed as the mean ± standard deviation (SD). The data were subjected to a one-way analysis of variance (ANOVA) followed by Duncan’s multiple-range test to determine significant differences among treatments using the SPSS 13.0 software package (SPSS Inc., Chicago, IL, USA). *P* < 0.05 was considered statistically significant. Parameters with significant differences were subjected to a second-degree polynomial regression analysis.

## 3. Results

### 3.1. Muscle composition and flesh quality parameters

The effects of different levels of dietary valine on product quality are given in Table 4 and S2 Table. Dietary valine did not significantly affect the 24-h post mortem pH of grass carp (*P* > 0.05). The relative shear force increased with increased dietary valine up to 16.7 g/kg and decreased thereafter (*P* < 0.05). The cooking loss was the highest for fish fed the basal diet and significantly decreased with increased dietary valine up to 13.1 g/kg (*P* < 0.05) and remained nearly constant thereafter (*P* > 0.05). The hydroxyproline content increased with increased dietary valine up to 10.6 g/kg and decreased with a further increase in dietary valine concentration (*P* < 0.05). Cathepsin B and L activities decreased with increased dietary valine concentration up to 13.1 g/kg and significantly increased thereafter (*P* < 0.05). The second-degree polynomial regression equations for the relationships of relative shear force, cooking loss and cathepsin B in grass carp muscle with the dietary valine level were as follows: Y_relative shear force_ = -0.003x^2^ + 0.083x + 1.641, R^2^ = 0.652, *P* = 0.205; Y_cooking loss_ = 0.130x^2^–3.067x + 30.496, R^2^ = 0.905, *P* < 0.05; and Y_cathepsin B_ = 0.007x^2^–0.196x + 2.822, R^2^ = 0.858, *P* = 0.054.

**Table 4:** The pH 24 h post mortem, relative shear force, cooking lose (%), hydroxyproline conten (μg/mg tissue), cathepsin B and L (U g^-1^ protein) in muscle of grass carp fed diets with graded levels of valine for 60 days.

	Dietary valine levels (g kg ^-1^ diet)					
	4.3	8.0	10.6	13.1	16.7	19.1
pH 24 h	6.08±0.02^a^	6.07±0.05^a^	6.08±0.04^a^	6.07±0.07^a^	6.09±0.04^a^	6.12±0.06^a^
Relative shear force	1.97±0.10^a^	2.07±0.11^ab^	2.14±0.14^bc^	2.19±0.07^cd^	2.28±0.08^d^	2.02±0.10^ab^
Cooking lose	19.4±1.35^d^	14.7±0.89^b^	13.7±0.59^b^	10.8±0.63^a^	16.0±0.98^c^	16.3±1.09^c^
Hydroxyproline	0.10±0.01^a^	0.14±0.01^cd^	0.15±0.01^d^	0.12±0.01^b^	0.14±0.01^cd^	0.13±0.01^bc^
Cathepsin B	2.02±0.16^d^	1.87±0.09^d^	1.47±0.14^bc^	1.28±0. 14^a^	1.37±0.14^ab^	1.55±0.15^c^
Cathepsin L	1.58±0.15^b^	1.53±0.17^b^	1.12±0.14^a^	1.18±0.08^a^	1.64±0.21^b^	1.65±0.20^b^

Values are means ± SD (n = 6), and different superscripts (a,b,c) in the same row are significantly different (*P* < 0.05).

The effects of the different levels of dietary valine on muscle composition are presented in Table 5 and S3 Table. The muscle protein and lipid contents significantly increased with increased dietary valine up to 13.1 g/kg (*P* < 0.05) and remained nearly constant thereafter (*P* > 0.05). However, the muscle calcium content significantly decreased with increased dietary valine up to 10.6 g/kg (*P* < 0.05) and remained nearly constant thereafter (*P* > 0.05). No significant differences in muscle moisture or ash content among the groups were evident (*P* > 0.05). Furthermore, the relationships of the proteins and lipids in grass carp muscle with the dietary level of valine were quadratic (Y_protein_ = -0.008x^2^ + 0.259x + 16.434, R^2^ = 0.776, *P* = 0.106; Y_lipid_ = -0.008x^2^ + 0.218x + 1.170, R^2^ = 0.869, *P <* 0.05).

**Table 5:** Muscle composition (g kg ^-1^) of grass carp fed diets with graded levels of valine for 60 days.

	Dietary valine levels (g kg ^-1^ diet)					
	4.3	8.0	10.6	13.1	16.7	19.1
Moisture	773.4±16.1^a^	769.1±6.4^a^	772.7±9.7^a^	761.3±5.1^a^	768.2±8.1^a^	764.2±4.3^a^
Protein	174.8±12.7^a^	179.2±7.0^abc^	180.5±9.3^abc^	189.6±6.4^c^	184.5±2.8^abc^	186.1±2.5^bc^
Lipid	19.7±2.2^a^	24.2±1.3^b^	25.1±1.6^b^	28.5±2.0^c^	25.1±1.5^b^	25.3±0.5^b^
Ash	13.7±0.9^a^	13.4±1.3^a^	13.5±1.6^a^	14.0±0.7^a^	13.2±0.5^a^	13.7±0.7^a^
Ca	2.4±0.4^b^	2.2±0.2^ab^	2.1±0.2 ^a^	2.1±0.2 ^a^	2.0±0.1 ^a^	2.1±0.2 ^a^
P	2.3±0.1^b^	2.3±0.2^ab^	2.2±0.2^ab^	2.2±0.1^ab^	2.1±0.1 ^a^	2.2±0.2^ab^

Values are means ± SD (n = 6), and different superscripts (a,b,c) in the same row are significantly different (*P* < 0.05).

The effects of different levels of dietary valine on the composition of protein-bound AAs in grass carp muscle are shown in Table 6. The concentrations of aspartic acid, threonine, cysteine, methionine, leucine, phenylalanine, lysine, and histidine in muscle significantly increased with the valine level up to 10.6, 8.0, 13.1, 13.1, 13.1, 8.0, 10.6, and 13.1 g/kg, respectively, and decreased thereafter (*P* < 0.05). The glutamine, tyrosine, arginine and valine contents significantly increased with dietary valine levels up to 8.0, 19.1, 13.1 and 13.1 g/kg, respectively, and remained nearly constant thereafter (*P* > 0.05). However, increasing the valine level in the diet did not significantly affect the concentration of serine, glycine, alanine or isoleucine (*P* > 0.05). Regression analyses showed that the concentrations of threonine, glutamine, methionine, leucine, phenylalanine, lysine, arginine and EAAs in the fillets had significant quadratic responses to the dietary valine level (Y_threonine_ = -0.006x^2^ + 0.114x + 2.868, R^2^ = 0.857, *P* = 0.054; Y_glutamine_ = -0.018x^2^ + 0.510x + 10.309, R^2^ = 0.866, *P* < 0.05; Y_methionine_ = -0.004x^2^ + 0.096x + 1.573, R^2^ = 0.816, *P* = 0.079; Y_leucine_ = -0.010x^2^ + 0.272x + 4.040, R^2^ = 0.832, *P* = 0.069; Y_phenylalanine_ = -0.004x^2^ + 0.068x + 3.082, R^2^ = 0.732, *P* = 0.138; Y_lysine_ = -0.016x^2^ + 0.312x + 8.811, R^2^ = 0.785, *P* = 0.100; Y_arginine_ = -0.009x^2^ + 0.254x + 3.387, R^2^ = 0.861, *P* = 0.052; Y_EAA_ = -0.064x^2^ + 1.507x + 27.315, R^2^ = 0.876, *P* < 0.05).

**Table 6:** Protein-bound amino acids composition of grass carp muscle (g/100 g dry).

	Dietary valine levels (g kg ^-1^ diet)					
	4.3	8.0	10.6	13.1	16.7	19.1
Aspartic acid	7.92±0.42^a^	7.83±0.14^a^	9.43±0.43^b^	7.73±0.38^a^	7.49±0.30^a^	7.50±0.08^a^
Threonine	3.22±0.25^ab^	3.54±0.12^b^	3.35±0.08^ab^	3.37±0.12^ab^	3.23±0.33^ab^	2.97±0.12^a^
Serine	3.18±0.17^a^	3.36±0.03^a^	3.61±0.36^a^	3.37±0.11^a^	3.25±0.13^a^	3.34±0.24^a^
Glutamine	12.0±0.12^a^	13.5±0.46^b^	13.8±1.00^b^	13.6±0.69^b^	13.5±0.49^b^	13.5±0.16^b^
Glycine	3.69±0.10^a^	3.70±0.07^a^	3.82±0.07^a^	3.61±0.05^a^	3.60±0.16^a^	3.71±0.07^a^
Alanine	4.14±0.11^a^	4.23±0.15^a^	4.20±0.01^a^	4.29±0.02^a^	4.02±0.47^a^	4.21±0.11^a^
Cystine	0.52±0.05^ab^	0.52±0.01^ab^	0.55±0.04^b^	0.55±0.04^b^	0.45±0.00^a^	0.52±0.05^ab^
Methionine	1.95±0.13^a^	2.02±0.08^ab^	2.16±0.03^b^	2.20±0.01^b^	2.14±0.07^ab^	1.96±0.02^a^
Isoleucine	3.37±0.20^a^	3.31±0.03^a^	3.31±0.12^a^	3.37±0.07^a^	3.33±0.15^a^	3.30±0.15^a^
Leucine	5.09±0.14^a^	5.34±0.18^ab^	5.87±0.12^c^	5.95±0.03^c^	5.60±0.08^b^	5.49±0.01^b^
Tyrosine	2.49±0.04^a^	2.71±0.20^ab^	2.64±0.06^ab^	2.72±0.04^ab^	2.69±0.14^ab^	2.83±0.01^b^
Phenylalanine	3.22±0.32^abc^	3.50±0.04^c^	3.42±0.03^bc^	3.06±0.05^ab^	3.03±0.14^ab^	2.90±0.14^a^
Lysine	6.83±0.24^abc^	7.09±0.51^bc^	7.65±0.76^c^	7.10±0.16^bc^	5.99±0.23^a^	6.06±0.21^ab^
Histidine	1.54±0.10^ab^	1.56±0.08^ab^	1.68±0.03^bc^	1.80±0.01^c^	1.44±0.03^a^	1.47±0.00^a^
Arginine	4.36±0.08^a^	4.68±0.28^ab^	5.32±0.35^b^	5.26±0.41^b^	5.11±0.08^b^	5.18±0.16^b^
Valine	3.31±0.09^a^	3.32±0.33^a^	3.81±0.02^ab^	4.13±0.48^b^	3.95±0.23^ab^	3.66±0.03^ab^
Total EAA	32.8±0.26^a^	34.4±0.10^b^	36.8±0.37^d^	36.2±0.15^c^	34.1±0.53^b^	32.8±0.25^a^

Values are means ± SD (n = 6), and different superscripts (a,b,c) in the same row are significantly different (*P* < 0.05).

The effects of different levels of dietary valine on the FA profile are shown in Table 7. The concentrations of C15:0, C16:1, C20:2, C20:5+C22:0 and saturated FAs (SFAs) in the fillets significantly decreased with an increase in the valine level up to 10.6, 16.7, 16.7, 13.1, and 16.7 g/kg, respectively (*P* < 0.05), and remained nearly constant thereafter (*P* > 0.05). However, the C16:0 and C18:0 contents in muscle significantly increased as the valine level increased up to 8.0 and 13.1 g/kg, respectively (*P* < 0.05). The C20:1, C18:1c+t, C18:3n-3 and unsaturated FA (UFA) contents; the monounsaturated fatty acid (MUFA) content; and the PUFA content in muscle significantly increased as the valine level increased up to 10.6, 16.7, 16.7, 16.7, 16.7 and 10.6 g/kg (P < 0.05), respectively, and increased as valine level further increased (*P* < 0.05). The following equations were obtained for C15:0, C16:0, C16:1, C20:2, C22:6, SFAs, UFAs and PUFAs in the fillets: Y_C15:0_ = -0.0004x^2^ + 0.0047X + 0.376, R^2^ = 0.850, *P* = 0.058; Y_C16:0_ = -0.050x2–1.245x + 32.655, R^2^ = 0.896, *P* < 0.05; Y_C16:1_ = -0.008x^2^–0.220x + 12.197, R^2^ = 0.832, *P* = 0.069; Y_C20:2_ = -0.0003x^2^–0.012x + 0.624, R^2^ = 0.910, *P* < 0.05; Y_C22:6_ = -0.015x^2^ + 0.374x + 8.312, R^2^ = 0.780, *P* = 0.103; Y_SFA_ = 0.052x^2^–1.273x + 42.004, R^2^ = 0.910, *P* < 0.05; Y_UFA_ = -0.052x^2^–1.272x + 57.639, R^2^ = 0.911, *P* < 0.05; and Y_PUFA_ = -0.023x^2^ + 0.152x + 19.964, R^2^ = 0.816, *P* = 0.079.

**Table 7:** Effect of dietary Valine on the fillet fatty acid (FA) profile (% of total FA methyl esters; mean values and standard deviation of individual fish, *n =* 3).

FA	Dietary Valine levels (g/kg diet)					
	4.3	8.0	10.6	13.1	16.7	19.1
C14:0	3.06±0.17^a^	3.09±0.07^a^	3.02±0.10^a^	3.00±0.30^a^	3.04±0.17^a^	3.05±0.08^a^
C15:0	0.32±0.01^c^	0.30±0.02^b^^c^	0.29±0.02^a^^b^^c^	0.26±0.04^a^^b^	0.26±0.01^a^	0.28±0.02^a^^b^^c^
C16: 0	27.9±0.69^c^	26.7±1.25^b^^c^	24.9±0.29^a^	24.6±0.35^a^	25.8±1.42^a^^b^	27.4±0.64^b^^c^
C17:0	0.25±0.02^a^	0.25±0.01^a^	0.24±0.02^a^	0.25±0.01^a^	0.23±0.01^a^	0.23±0.01^a^
C18:0	4.60±0.12^a^^b^	4.50±0.08^a^	4.61±0.08^a^^b^	4.93±0.10^c^	4.84±0.25^b^^c^	4.95±0.10^c^
C20:0	0.26±0.01^a^	0.26±0.01^a^	0.25±0.02^a^	0.26±0.00^a^	0.24±0.02^a^	0.25±0.01^a^
C23:0	0.13±0.01^a^	0.12±0.01^a^	0.13±0.01^a^	0.13±0.01^a^	0.12±0.00^a^	0.12±0.00^a^
C24:0	0.68±0.02^a^	0.69±0.03^a^	0.64±0.02^a^	0.64±0.00^a^	0.66±0.05^a^	0.65±0.02^a^
C14:1	0.20±0.01^a^	0.20±0.02^a^	0.19±0.02^a^	0.18±0.00^a^	0.18±0.01^a^	0.18±0.02^a^
C16:1	13.0±0.40^a^	13.4±0.45^a^^b^	13.8±0.27^b^	13.7±0.48^a^^b^	13.5±0.42^a^^b^	13.6±0.48^a^^b^
C17:1	0.41±0.03^a^	0.41±0.02^a^	0.41±0.03^a^	0.41±0.03^a^	0.40±0.02^a^	0.42±0.03^a^
C18:1c+t	25.4±1.28^a^^b^	25.1±1.18^a^^b^	25.6±0.53^a^^b^	26.6±0.67^b^	26.3±0.56^a^^b^	24.8±0.43^a^
C18:2c+t	5.86±0.18^a^	6.03±0.29^a^	6.13±0.14^a^	6.29±0.29^a^	6.02±0.14^a^	5.97±0.40^a^
C18:3n-6	0.29±0.03^a^	0.32±0.04^a^	0.29±0.03^a^	0.30±0.01^a^	0.29±0.03^a^	0.29±0.01^a^
C18:3n-3	1.25±0.04^a^^b^	1.21±0.06^a^	1.34±0.07^b^	1.33±0.05^b^	1.33±0.05^b^	1.20±0.05^a^
C20:1	1.84±0.03^a^	1.94±0.18^a^^b^	2.17±0.17^b^	1.95±0.11^a^^b^	1.73±0.13^a^	1.73±0.06^a^
C20:2	0.57±0.01^b^	0.56±0.04^a^^b^	0.51±0.02^a^^b^	0.51±0.06^a^^b^	0.49±0.05^a^	0.49±0.03^a^^b^
C20:3n-6+C21:0	0.29±0.01^a^	0.30±0.01^a^	0.30±0.01^a^	0.29±0.01^a^	0.29±0.02^a^	0.30±0.00^a^
C20:3n-3	2.51±0.14^a^	2.58±0.11^a^	2.67±0.13^a^	2.61±0.14^a^	2.61±0.14^a^	2.62±0.10^a^
C20:4	0.36±0.02^a^	0.35±0.00^a^	0.35±0.01^a^	0.35±0.01^a^	0.35±0.02^a^	0.34±0.03^a^
C20:5+C22:0	1.10±0.03^b^	1.12±0.08^b^	1.11±0.06^b^	0.83±0.12^a^	0.84±0.09^a^	0.86±0.02^a^
C22:1	0.18±0.00^a^	0.18±0.02^a^	0.18±0.00^a^	0.18±0.01^a^	0.17±0.01^a^	0.17±0.01^a^
C22:6	9.58±0.45^a^	10.37±0.25^b^^c^	10.93±0.17^c^	10.41±0.38^b^^c^	10.28±0.36^b^	10.17±0.23^b^
SFA^a^	37.2±0.82^c^	35.9±1.26^b^^c^	34.1±0.16^a^	34.1±0.69^a^	35.2±1.50^a^^b^	36.9±0.49^b^^c^
UFA^b^	62.4±0.84^a^	63.7±1.25^a^^b^	65.6±0.17^c^	65.5±0.71^c^	64.4±1.51^b^^c^	62.7±0.50^a^^b^
MUFA^c^	41.0±0.93^a^	41.2±1.30^a^^b^	42.3±0.51^a^^b^	43.0±1.12^b^	42.3±0.77^a^^b^	40.9±0.71^a^
PUFA^d^	21.8±0.25^a^	22.9±0.05^b^^c^	23.6±0.41^c^	22.9±0.42^b^^c^	22.5±0.74^a^^b^	22.2±0.39^a^^b^

^a^Saturated fatty acid

^b^Unsaturated fatty acid

^c^Monounsaturated fatty acid

^d^Polyunsaturated fatty acids

### 3.2. Muscle antioxidant parameters

The contents of MDA and PC and the activities of SOD1, CAT, Se-GPx, and GSH in grass carp muscle fed different levels of valine are shown in Table 8 and S4 Table. The MDA and PC contents significantly decreased as dietary valine increased up to 13.1 g/kg and increased thereafter (*P* < 0.05). The CAT and SOD1 activities significantly increased when the dietary valine level was 13.1 g/kg and gradually decreased thereafter (*P* < 0.05). The GSH content gradually increased with dietary valine levels from 4.3 to 10.6 g/kg and significantly decreased thereafter (*P* < 0.05). The Se-GPx activity increased as dietary valine level increased up to 16.7 g/kg and remained nearly constant thereafter (*P* > 0.05). Furthermore, the MDA, PC, and GSH contents and the Se-GPx activities in grass carp muscle had significant quadratic responses to increased dietary valine levels. A regression analysis showed that the muscle MDA and PC contents and GSH and Se-GPx activities had quadratic responses to the changes in dietary valine (Y_MDA_ = 0.010x^2^–0.305x + 3.647, R^2^ = 0.940, *P* < 0.05; Y_PC_ = 0.033x^2^–0.966x + 9.344, R^2^ = 0.936, *P* < 0.05; Y_GSH_ = -0.045x^2^ + 0.962x + 4.107, R^2^ = 0.698, *P* = 0.166; Y_Se-GPx_ = -0.083x^2^ + 3.261x + 92.677, R^2^ = 0.688, *P* = 0.175).

**Table 8:** Malondialdehyde content (MDA, nmol mg^-1^ protein), protein carbonyl content (PC, nmol mg^-1^ protein), activities of superoxide dismutase 1 (SOD1, U mg^-1^ protein), catalase (CAT, U mg^-1^ protein), Selenium-dependent glutathione peroxydase (Se-GPx, U mg^-1^ protein), and reduced glutathione content (GSH, mg g^-1^ protein) content in muscle of juvenile grass carp fed diets containing graded levels of valine for 60 days.

	Dietary valine levels (g kg ^-1^ diet)					
	4.3	8.0	10.6	13.1	16.7	19.1
MDA	2.57±0.16^d^	1.73±0.14^c^	1.71±0.02^c^	1.35±0.09^a^	1.53±0.07^b^	1.55±0.13^b^
PC	5.71±0.41^f^	3.72±0.32^e^	3.30±0.21^d^	1.74±0.16^a^	2.40±0.18^b^	2.90±0.27^c^
SOD1	2.82±0.26^a^	3.08±0.19^ab^	3.47±0.26^cd^	4.46±0.29^e^	3.69±0.21^d^	3.23±0.30^bc^
CAT	1.21±0.06^a^	1.35±0.13^ab^	1.57±0.15^c^	2.91±0.18^d^	1.52±0.14^c^	1.41±0.41^bc^
GSH	7.62±0.54^b^	7. 83±0.56^bc^	10.61±0.90^d^	8.48±0.65^c^	7.25±0.54 ^b^	6.09±0.60^a^
Se-GPx	108.7±11.01^ab^	107.0±11.12^a^	116.3±9.82^abc^	124.5±11.59^bc^	130.0±11.05^c^	120.1±7.88^abc^

Values are means ± SD (n = 6), and different superscripts (a,b,c) in the same row are significantly different (*P* < 0.05).

### 3.3. Relative expression of the antioxidant enzyme genes Nrf2, Keap1, TOR and S6K1 in muscle

**Fig 1:**
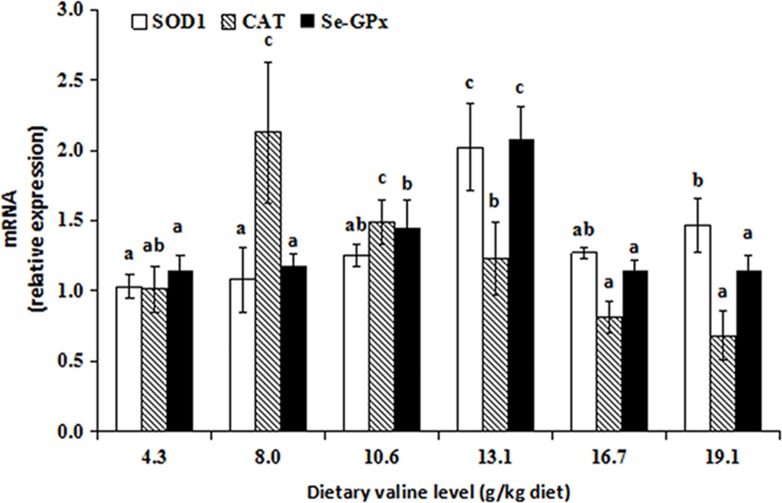
Effects of dietary valine on copper/zinc superoxide dismutase (SOD1), catalase (CAT) and Selenium-dependent glutathione peroxydase (Se-GPx) expression in grass carp muscle. Values are means ± SD (n = 6), and different letters denote the significant difference (*P* < 0.05).

**Fig 2:**
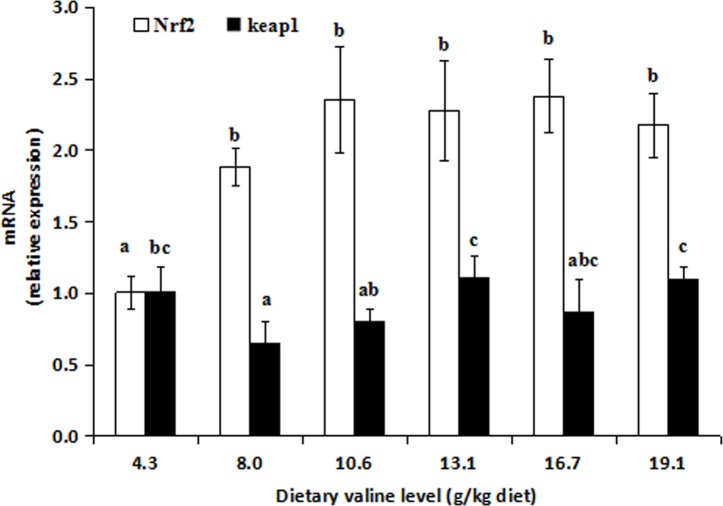
Effects of dietary valine on NF-E2-related factor 2(Nrf2) and Kelch-like- ECH-associated protein 1(keap1) expression in grass carp muscle. Values are means ± SD (n = 6), and different letters denote the significant difference (*P* < 0.05).

**Fig 3:**
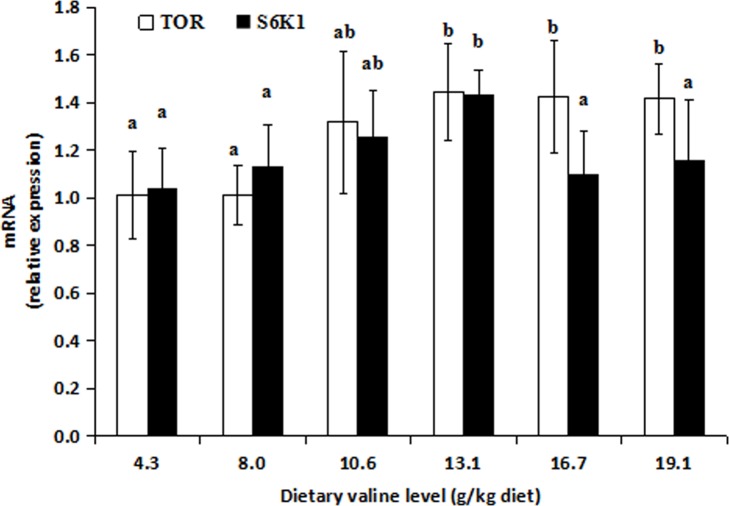
Effects of dietary valine on target of rapamycin (TOR) and S6 Kinase 1(S6K1) expression in grass carp muscle. Values are means ± SD (n = 6), and different letters denote the significant difference (*P* < 0.05).”

The effects of dietary valine on the relative expression of the SOD1, CAT, Se-GPx, Nrf2, Keap1, TOR and S6K1 genes in the muscle are presented in Figs 1–3 and S5 Table. The SOD1 and Se-GPx mRNA levels significantly increased as dietary valine level increased up to 13.1 g/kg (*P* < 0.05) and then decreased thereafter (*P* < 0.05). The relative expression of the CAT gene significantly increased as dietary valine level increased up to 8.0 g/kg (*P* < 0.05) and then decreased thereafter (*P* < 0.05). As shown in Figs 2 and 3, the Nrf2 mRNA level increased as dietary valine level increase up to 8.0 g/kg (*P* < 0.05) and then remained nearly constant thereafter (*P* > 0.05). The Keap1 mRNA level remarkably decreased as dietary valine level increased up to 10.6 g/kg (*P* < 0.05) and then increased as dietary valine level increased from 13.1 to 19.1 g/kg (*P* > 0.05). The TOR and S6K1 mRNA levels were the lowest in fish fed the basal diet and the highest in fish fed 13.1 g/kg valine (*P* < 0.05). The relationships of the CAT and TOR mRNA levels with the dietary valine level were quadratic: Y_CAT_ = -0.005x^2^ + 0.153x + 0.399, R^2^ = 0.790, *P* = 0.096 and Y_TOR_ = -0.002x^2^ + 0.085x + 0.624, R^2^ = 0.832, *P* = 0.069.

## 4. Discussion

As previously mentioned, flesh quality deterioration is one of the most important issues in the aquaculture industry and a major concern for consumers [12]. An earthy and musty odor or taste in fish can cause a major reduction in consumption or make a product unsuitable for sale [3]. Nutrition has been suggested as a safe way to improve fish flesh quality and thus preservation quality and consumer acceptance [4]. Our previous study indicated that valine could improve the growth and development of grass carp intestines [12] and gills [13], which are associated with its antioxidant defenses [14]. Gan et al. [8] suggested that deteriorated flesh quality was related to the destruction of muscle antioxidant defense systems. These observations led us to hypothesize that valine might also influence the flesh quality of fish. Therefore, we investigated the effects of valine on the flesh quality of young grass carp.

### 4.1. Valine improved the physical quality of young grass carp fillets

Mouthfeel is the most important attribute in the evaluation of fish quality and can be evaluated by relative shear force and WHC [9–11]. Bahuaud et al. [10] reported that soft flesh decreases the acceptability and quality of fish meat. Shear force is a biomarker that represents the firmness of fish flesh [52]. The present study showed that dietary valine significantly increases the relative shear force of young grass carp muscle, suggesting that valine could increase fish muscle firmness. The positive effects of dietary valine on the firmness of fish may be partly explained by the effects of an optimum level of dietary valine on collagen synthesis and on the cathepsin B and L activities. In plaice (*Pleuronectes platessa*), a high collagen content results in flesh firmness [53]. Hydroxyproline is essential for the synthesis of collagen in fish meat, and hydroxyproline content has been used to calculate collagen content [54]. The present study showed that dietary valine significantly increases the hydroxyproline content of fish muscle, following a similar pattern as relative shear force, indicating that valine can increase fish muscle firmness by increasing collagen content. In addition, cathepsin B and L play a key role in post-mortem muscle softening [55]. Bahuaud et al. [52] reported that decreased firmness in Atlantic salmon (*Salmo salar* L.) is partly attributable to increased muscle cathepsin B and L activities. In our study, the activities of cathepsin B and L in the muscle decreased as dietary valine levels increased, and a correlation analysis showed that the activity of cathepsin B was negatively correlated with the relative shear force (r = - 0.802, *P* = 0.055), suggesting that the increase in fish muscle firmness due to valine may be partly explained by the decrease in cathepsin B activity due to valine. However, this mechanism must be further investigated. In addition to firmness, a low WHC has a detrimental impact on technological quality and sensory quality for the consumer [6], as reflected by the cooking loss in fish meat [56]. Our results demonstrated that less cooking loss occurs in grass carp muscle when dietary valine is supplemented to an optimum level, indicating that valine could enhance the WHC in the muscle. The beneficial effects of dietary valine on the muscle WHC may be partly explained by the positive effect of valine on the antioxidant capacity. Another study in chicks showed that increased muscle WHC can be attributed to the protection of muscle structural integrity resulting from decreased oxidative damage [57]. In fish, valine is a major substrate for the synthesis of α-MSH [9]. In both keratinocytes and melanocytes, α-MSH significantly improves the antioxidant status and increases γ-glutamylcysteine-synthetase and GSH-S-transferase Pi gene expression [10]. Valine inhibits deoxyribose attack *in vitro* by •OH, which is induced by Fenton-like reactions [11]. Recent studies in fish have suggested that cooking loss [8] is sensitive indicators of the dietary valine requirement in fish muscle. The dietary valine requirements of young grass carp (268–679 g) as estimated from muscle cooking loss using quadratic regression were 11.8 g/kg diet, corresponding to 39.3 g/kg protein, which was lower than the valine requirement for percent weight gain in grass carp (14.00 g/kg diet, corresponding to 47.7 g/kg protein) [12], suggesting that nutrient requirements may vary with sensitive indicators. This phenomenon may be explained by the function of valine in protein deposition in muscle tissue [58]. However, the underlying mechanism requires further investigation.

In addition to mouthfeel, flavor and FA profile are two important flesh quality parameters that affect consumer perception [15]. Flavor components are formed by the reactions of AAs, which are the basic units of proteins [16, 17]. In the present study, the muscle protein content was significantly higher at the optimum dietary valine level. FAs are carboxylic acids with long aliphatic chains, which are the important parts of lipids [59]. Our study showed that the fish muscle lipid content increased as dietary valine levels increased. These data suggest that valine may affect flesh quality by improving the flavor and FA profile of the fillet and requires further investigation.

### 4.2. Valine improved the flavor of young grass carp fillets

Fish flavor is an important flesh quality characteristic, and a musty odor could lead to poor market value and consumption [15]. A study reported that the odors obtained from these reactions depend on the AAs present [16]. Research has shown that AAs such as glutamate, aspartic acid, glycine, alanine and histidine contribute directly to flavor development [16, 17]. The present study showed that valine increased the protein-bound AAs in muscle such as aspartic acid, threonine, glutamine, cystine, methionine, leucine, tyrosine, phenylalanine, lysine, histidine, arginine and valine contents, suggesting that valine may have partially improved the flavor of young grass carp fillets by altering the AA concentration in the fish muscle. The positive effects of including an optimum level of dietary valine on the AA concentration might be related to the EAA status and the Na^+^, K^+^-ATPase and AA balance. The levels of specific EAAs in fish muscle have been used as an index of dietary AA status [60]. In the present study, the total grass carp muscle EAA content significantly increased with 10.6 g/kg valine supplementation. In addition, a better balance of AA in the diet reduces the PAC in fish [19]. We previously found that valine decreases the PAC of grass carp [12], suggesting that an optimum valine level could balance AA content and promote protein synthesis. Moreover, AA absorption requires an extracellular sodium ion concentration gradient [61]. To our knowledge, Na^+^, K^+^-ATPase is located on the basolateral membrane and can transport Na^+^ out of the cell [21]. In juvenile Jian carp (*Cyprinus carpio var*. Jian), optimum valine supplementation increased Na^+^, K^+^-ATPase activity [22].

Interestingly, the data from this study show that dietary valine has no effect on the concentrations of aspartic acid, serine, glycine and isoleucine. Aspartic acid, serine, and glycine are non-EAAs and can be synthesized *in vivo* [62]. Thus, the negligible effects of valine on the concentrations of aspartic acid, serine, and glycine may occur because these AAs are synthesized *de novo* by the organism. In addition, the absorption of isoleucine and valine involves the BCAA transport system [63], which has antagonistic effects in terrestrial animals [64]. Szmelcman et al. [65] reported that isoleucine strongly inhibits the absorption rate of valine in rats. Therefore, the negligible effect of valine on isoleucine may be partly related to antagonism between isoleucine and valine. However, the underlying mechanism requires further study. In addition to the flavor of young grass carp fillets, the FA profile is a key flesh quality characteristic that has been implicated in diseases, including various cancers and coronary heart disease [66]. Thus, we next investigated the effects of valine on the FA composition of the muscle of young grass carp.

### 4.3. Valine improved the fatty acid profile of young grass carp fillets

In this study, optimal valine supplementation decreased the SFA (C15:0 and C16:0) concentration and increased the UFA (MUFAs, such as C16:1, C18:1c+t and C20:1, and PUFAs such as C18:3n-3, C20:2 and C22:6) concentration, suggesting that valine enhanced the potential health benefits of fish by increasing the SFA and UFA concentrations in fish muscle. Valine may have improved the FA profile through its positive effects on nutrient digestibility and regulation of FA synthesis. Lipase is an enzyme that catalyzes the hydrolysis of fats and is involved in the synthesis of FAs [67]. A recent study in our laboratory indicated that valine increases lipase activity in the hepatopancreas and intestines of fish [22]. In addition, FAS is a critical enzyme for lipogenesis in mammals [24]. Du et al. [26] reported that valine increases FAS expression in mouse liver.

Additionally, in the present study, it is interesting that excess valine significantly negatively affected the deposition of nutrients (decreased hydroxyproline, protein, lipid, threonine, methionine, leucine, phenylalanine, lysine, histidine, C18:1c+t, C18:3n-3, C20:1, C20:5+C22:0, and C22:6 concentrations) in the muscle of grass carp. These observations indicate the negative effect of excess valine on nutrient deposition in fish muscle. The reasons for this effect are unknown, but there are two possible explanations. First, as mention above, BCAAs share the same transporter on the cell membrane [63], and imbalances in their dietary composition can result in impaired nutrient absorption [68]. Second, excess nutrient delivery can increase the metabolic demand of acute injury and place additional burden on the tissue, leading to dysfunction of tissue *in vivo* [69]. Thus, the negative effects of high doses of valine on the deposition of nutrients may be attributed to AA imbalance and excess tissue burden, but the underlying mechanism requires further investigation. Improved fillet quality in fish is positively related to antioxidant defenses [4]. As mentioned above, dietary valine improves the fillet quality of fish, leading us to hypothesize that valine might have beneficial effects on antioxidant defense; thus, we next investigated the effects of valine on antioxidant defenses in grass carp muscle.

### 4.4. Valine improved the antioxidant defenses in young grass carp muscle

In our study, the MDA and PC contents in fish muscle were lower under valine supplementation, suggesting that lipid peroxidation and protein oxidation are suppressed by valine in fish muscle. Lipid peroxidation and protein oxidation in aquatic organisms result from excessive ROS production [70], and ROS can rapidly be removed by non-enzymatic antioxidants, such as reduced GSH and the antioxidant enzymes SOD1, CAT and Se-GPx [27]. Therefore, we also determined the effects of valine on the GSH content and the activities of SOD1, CAT and Se-GPx. The GSH content in the muscle significantly increased as the dietary valine level increased. No previous report has investigated the effects of valine on the GSH content in fish. The mechanism by which valine increased the GSH content in the present study and previously in intestinal epithelial cells of juvenile Jian carp [71] may be related to the role of valine in porcine enterocytes as a substrate for glutamine synthesis [72]. In addition, our results showed that the muscle SOD1, CAT and Se-GPx activities increase with valine supplementation. However, no information about the effects of valine on the activities of these antioxidant enzymes in fish is available. The positive effects of valine on the SOD1, CAT and Se-GPx activities may be partly explained by the positive effect of valine on the mRNAs of those antioxidant enzymes. We previously reported that increased enzyme activity in fish is correlated with enhanced transcription [73]. In this study, with increased valine supplementation, the SOD1, CAT and Se-GPx mRNA levels in the muscle increased and followed a pattern similar to their respective changes in enzyme activity. Additionally, correlation analyses showed that the SOD1, CAT and Se-GPx activities were positively related to their respective mRNA levels (r_1_ = + 0.868, *P*_*1*_ < 0.05; r_2_ = + 0.812, *P*_*2*_ = 0.05; r_3_ = + 0.623, *P*_*3*_ = 0.186), supporting the hypothesis that the mechanism by which valine increases the activities of SOD1, CAT and Se-GPx is related to the up-regulation of the mRNA levels of these genes. However, no data exist concerning the effects of valine on the expression of SOD1, CAT and Se-GPx in fish. The Nrf2-Keap1 signaling pathway in fish plays a key role in the expression of antioxidant enzyme genes [30]. Thus, we next investigated the relationship between valine and the Nrf2-Keap1 signaling pathway in young grass carp.

### 4.5. Valine regulated Nrf2, Keap1, TOR and S6K1 gene expression in the muscle of young grass carp

Nrf2 regulates the induction of genes encoding antioxidant enzymes, including SOD1, CAT and Se-GPx [30]. In mice, improved Nrf2 mRNA levels can up-regulate SOD1 [74], Se-GPx [75] and CAT [76] expression. In this study, Nrf2 expression in the muscle of young grass carp was up-regulated as the dietary valine level increased. A correlation analysis indicated that the SOD1 and CAT mRNA levels were positively related to Nrf2 expression (r_1_ = + 0.641, *P*_*1*_ = 0.17; r_2_ = + 0.805, *P*_*2*_ = 0.054), suggesting that the increased antioxidant enzyme gene expression due to valine supplementation may be partly explained by the positive effect of valine on the Nrf2 mRNA level in fish. No study has investigated the beneficial effects of dietary valine on Nrf2 gene expression in fish. The effects of valine on the regulation of Nrf2 expression may be attributed to valine’s regulation of the TOR pathway. S6K1 is the major downstream target of TOR [31]. Up-regulated mTOR [77] and S6K1 [32] expression increases Nrf2 expression in mammals. The results of our current study demonstrate that the mRNA levels of TOR and S6K1 in the muscle increase with dietary valine levels, and a correlation analysis showed that the Nrf2 mRNA level is positively related to TOR (r = + 0.811, *P* = 0.005) in the muscle, indicating that the valine-induced increase in Nrf2 expression in fish may be partly due to the up-regulation of the TOR pathway. In addition, the positive effects of valine on antioxidant enzyme expression may be partly ascribed to an increase in Nrf2 nuclear translocation. Keap1 is a negative regulator of Nrf2 that sequesters Nrf2 in the cytoplasm and thereby prevents its nuclear translocation [30]. In mouse liver, the down-regulation of Keap1 expression could significantly increase Nrf2 nuclear translocation, resulting in the inducible expression of antioxidant genes [78]. In the present study, Keap1 expression decreased as dietary valine levels increased up to a certain level in muscle, suggesting that the valine-induced increase in the expression of antioxidant enzyme genes may be partly attributed to increased Nrf2 nuclear translocation caused by the down-regulation of Keap1 expression. However, the detailed mechanism requires further investigation.

## 5. Conclusions

In summary, our study has five main findings. (1) Dietary valine improves the physical characteristics of fish fillets (i.e., it increases the relative shear force and the hydroxyproline, protein and lipid contents and decreases the cathepsin B and L activities and cooking loss). (2) Valine improves the flavor of young grass carp fillets by increasing the concentrations of the AAs aspartic acid, threonine, glutamine, cystine, methionine, leucine, tyrosine, phenylalanine, lysine, histidine, arginine and valine. (3) Optimal valine supplementation enhances the potential health benefits of fish by decreasing the SFA concentration (C15:0 and C16:0) and increasing the UFA (MUFAs: C16:1, C18:1c+t and C20:1; PUFAs: C18:3n-3, C20:2 and C22:6) concentration. (4) Valine supplementation significantly improves the non-enzymatic and enzymatic status in the muscle of young grass carp. Moreover, SOD1, CAT, Se-GPx Nrf2, Keap1, TOR and S6K1 gene expression might further indicate the mechanism of the valine-induced improvement in antioxidant status in fish. (5) Interestingly, dietary valine has no effect on the concentrations of aspartic acid, serine, glycine or isoleucine. The amount of valine required based on percent weight gain was higher than that lost in cooking. However, additional studies should be carried out to determine the specific mechanisms by which valine produces these effects in fish.

## Supporting Information

S1 Table The size of the fish sampled (g fish^-1^) (n = 15). (DOCX)

S2 Table The pH 24 h post mortem, relative shear force, cooking lose (%), hydroxyproline conten (μg/mg tissue), cathepsin B and L (U g^-1^ protein) in muscle of grass carp supplemented with 4.3, 8.0, 10.6, 13.1, 16.7 and 19.1 g/kg valine (groups 1–6) for 60 days (n = 6). (DOCX)

S3 Table Muscle composition (g kg^-1^) of grass carp supplemented with 4.3, 8.0, 10.6, 13.1, 16.7 and 19.1 g/kg valine (groups 1–6) for 60 days (n = 6). (DOCX)

S4 Table Malondialdehyde content (MDA, nmol mg^-1^ protein), protein carbonyl content (PC, nmol mg^-1^ protein), activities of superoxide dismutase 1 (SOD1, U mg^-1^ protein), catalase (CAT, U mg^-1^ protein), and reduced glutathione (GSH, mg g^-1^ protein) content of grass carp supplemented with 4.3, 8.0, 10.6, 13.1, 16.7 and 19.1 g/kg valine (groups 1–6) for 60 days (n = 6). (DOCX)

S5 Table Copper/zinc superoxide dismutase (SOD1), catalase (CAT) and Selenium-dependent glutathione peroxydase (Se-GPx), NF-E2-related factor 2(Nrf2) and Kelch-like- ECH-associated protein 1(keap1), target of rapamycin (TOR) and S6 Kinase 1(S6K1) CT value of grass carp supplemented with 4.3, 8.0, 10.6, 13.1, 16.7 and 19.1 g/kg valine (groups 1–6) for 60 days (n = 6). (DOCX)
