# Supplementary material for: Physical and Flavor Characteristics, Fatty Acid Profile, Antioxidant Status and Nrf2-Dependent Antioxidant Enzyme Gene Expression Changes in Young Grass Carp (Ctenopharyngodon idella) Fillets Fed Dietary Valine
Source: PLoS One. 2017 Jan 24;12(1):e0169270. doi: 10.1371/journal.pone.0169270 (PMC5261571; doi:10.1371/journal.pone.0169270)
Supplement: S1 Table — (DOCX) [file pone.0169270.s001.docx]

**S1 Table.** The size of the fish sampled (g fish^-1^) (n=15).

|  | Dietary valine levels (g kg ^-1^ diet) | | | | | |
| --- | --- | --- | --- | --- | --- | --- |
|  | 4.3 | 8.0 | 10.6 | 13.1 | 16.7 | 19.1 |
|  | 361.68 | 501.22 | 600.30 | 670.80 | 598.44 | 576.98 |
| The size of the fish sampled (g fish^-1^) | 365.56 | 494.31 | 623.43 | 674.13 | 592.62 | 577.31 |
|  | 357.01 | 480.19 | 605.90 | 681.29 | 590.79 | 577.30 |
|  | 366.63 | 485.91 | 620.77 | 656.83 | 589.55 | 582.33 |
|  | 355.47 | 507.67 | 640.47 | 680.84 | 593.36 | 580.53 |
|  | 355.76 | 492.95 | 611.90 | 687.51 | 595.02 | 578.00 |
|  | 365.73 | 502.01 | 600.85 | 711.46 | 591.46 | 578.43 |
|  | 366.29 | 494.85 | 594.44 | 659.65 | 600.07 | 578.15 |
|  | 368.23 | 466.15 | 596.96 | 706.68 | 596.74 | 576.57 |
|  | 350.94 | 514.62 | 677.12 | 690.71 | 590.42 | 581.18 |
|  | 355.94 | 503.22 | 636.17 | 673.36 | 587.37 | 578.20 |
|  | 352.04 | 523.16 | 586.86 | 675.53 | 596.56 | 578.93 |
|  | 361.18 | 490.67 | 630.18 | 698.25 | 591.16 | 579.55 |
|  | 355.24 | 518.95 | 581.69 | 651.32 | 589.93 | 579.05 |
|  | 364.90 | 493.27 | 601.30 | 677.98 | 594.31 | 580.00 |
