# Supplementary material for: Physical and Flavor Characteristics, Fatty Acid Profile, Antioxidant Status and Nrf2-Dependent Antioxidant Enzyme Gene Expression Changes in Young Grass Carp (Ctenopharyngodon idella) Fillets Fed Dietary Valine
Source: PLoS One. 2017 Jan 24;12(1):e0169270. doi: 10.1371/journal.pone.0169270 (PMC5261571; doi:10.1371/journal.pone.0169270)
Supplement: S2 Table — (DOCX) [file pone.0169270.s002.docx]

**S2 Table.** The pH 24 h post mortem, relative shear force, cooking lose (%) , hydroxyproline conten (μg/mg tissue) , cathepsin B and L (U g^-1^ protein) in muscle of grass carp supplemented with 4.3, 8.0, 10.6, 13.1, 16.7 and 19.1 g/kg valine (groups 1-6) for 60 days (n=6).

| Number of groups | pH24 | Relative shear force | Cooking lose | Hydroxyproline conten | | Cathepsin B | | Cathepsin L | |
| --- | --- | --- | --- | --- | --- | --- | --- | --- | --- |
|  |  |  |  | weight of the sample | OD | estimated value | control value | estimated value | control value |
| 1-1 | 6.177 | 1.960 | 25.370 | 0.116 | 0.130 | 5.531 | 3.130 | 3.423 | 5.442 |
| 1-2 | 6.147 | 1.920 | 21.150 | 0.091 | 0.121 | 5.057 | 3.406 | 3.416 | 5.716 |
| 1-3 | 6.067 | 2.140 | 17.190 | 0.100 | 0.145 | 4.931 | 3.059 | 3.267 | 5.198 |
| 1-4 | 6.077 | 1.710 | 18.550 | 0.082 | 0.127 | 5.175 | 3.791 | 3.268 | 5.199 |
| 1-5 | 6.050 | 1.860 | 18.000 | 0.103 | 0.131 | 5.318 | 3.432 | 3.505 | 4.858 |
| 1-6 | 6.263 | 2.050 | 17.500 | 0.111 | 0.141 | 7.109 | 3.659 | 3.567 | 5.723 |
| 2-1 | 6.080 | 1.960 | 14.490 | 0.110 | 0.159 | 5.380 | 3.391 | 3.365 | 5.720 |
| 2-2 | 6.087 | 2.460 | 15.220 | 0.099 | 0.167 | 4.811 | 2.621 | 3.303 | 5.508 |
| 2-3 | 6.020 | 2.160 | 14.490 | 0.089 | 0.156 | 5.233 | 3.128 | 3.280 | 4.933 |
| 2-4 | 6.287 | 2.390 | 16.670 | 0.106 | 0.171 | 5.384 | 3.309 | 3.366 | 5.289 |
| 2-5 | 6.080 | 1.760 | 16.050 | 0.097 | 0.166 | 5.810 | 3.246 | 4.962 | 6.730 |
| 2-6 | 6.080 | 1.760 | 13.330 | 0.106 | 0.161 | 6.282 | 3.770 | 3.315 | 4.869 |
| 3-1 | 6.127 | 1.920 | 15.490 | 0.096 | 0.168 | 7.143 | 2.903 | 4.737 | 6.629 |
| 3-2 | 6.140 | 2.260 | 15.870 | 0.097 | 0.172 | 6.898 | 2.983 | 3.962 | 5.878 |
| 3-3 | 6.233 | 1.760 | 18.060 | 0.118 | 0.176 | 5.447 | 3.195 | 3.412 | 6.400 |
| 3-4 | 6.117 | 2.260 | 13.440 | 0.097 | 0.161 | 5.732 | 3.487 | 3.281 | 6.358 |
| 3-5 | 6.067 | 1.900 | 13.240 | 0.114 | 0.182 | 6.732 | 3.578 | 3.182 | 6.007 |
| 3-6 | 6.153 | 2.260 | 10.890 | 0.114 | 0.159 | 5.443 | 4.068 | 3.308 | 6.106 |
| 4-1 | 6.087 | 2.130 | 12.050 | 0.095 | 0.148 | 5.610 | 3.572 | 3.437 | 5.781 |
| 4-2 | 6.223 | 1.790 | 12.680 | 0.082 | 0.142 | 5.247 | 3.318 | 3.021 | 5.386 |
| 4-3 | 6.053 | 1.870 | 10.110 | 0.112 | 0.166 | 5.583 | 3.251 | 3.027 | 6.003 |
| 4-4 | 6.083 | 2.260 | 18.270 | 0.110 | 0.161 | 5.899 | 3.370 | 2.950 | 5.692 |
| 4-5 | 6.120 | 2.260 | 15.520 | 0.081 | 0.145 | 6.163 | 3.386 | 3.263 | 6.310 |
| 4-6 | 6.047 | 1.960 | 9.380 | 0.098 | 0.160 | 5.913 | 3.509 | 3.315 | 5.924 |
| 5-1 | 6.153 | 1.940 | 16.390 | 0.112 | 0.157 | 7.523 | 4.103 | 3.148 | 6.400 |
| 5-2 | 6.083 | 2.270 | 17.280 | 0.110 | 0.159 | 4.970 | 2.770 | 3.295 | 6.103 |
| 5-3 | 6.147 | 2.130 | 16.250 | 0.100 | 0.170 | 7.404 | 3.573 | 3.269 | 6.599 |
| 5-4 | 6.133 | 2.260 | 15.710 | 0.085 | 0.135 | 8.156 | 3.332 | 3.698 | 6.344 |
| 5-5 | 6.123 | 2.260 | 13.100 | 0.120 | 0.172 | 7.102 | 3.404 | 3.299 | 5.639 |
| 5-6 | 6.187 | 2.460 | 16.920 | 0.108 | 0.155 | 5.920 | 3.440 | 3.508 | 5.604 |
| 6-1 | 6.157 | 1.970 | 15.190 | 0.120 | 0.160 | 6.762 | 3.256 | 4.129 | 6.822 |
| 6-2 | 6.250 | 2.220 | 12.680 | 0.103 | 0.163 | 4.651 | 3.097 | 3.071 | 6.605 |
| 6-3 | 6.047 | 2.130 | 15.250 | 0.094 | 0.139 | 5.380 | 2.776 | 3.654 | 6.226 |
| 6-4 | 6.030 | 1.670 | 11.540 | 0.104 | 0.141 | 5.545 | 3.023 | 2.943 | 5.384 |
| 6-5 | 6.060 | 1.910 | 10.450 | 0.102 | 0.149 | 6.480 | 3.310 | 3.308 | 6.270 |
| 6-6 | 6.113 | 2.250 | 17.750 | 0.100 | 0.136 | 5.768 | 3.000 | 3.218 | 5.873 |
