# Supplementary material for: Physical and Flavor Characteristics, Fatty Acid Profile, Antioxidant Status and Nrf2-Dependent Antioxidant Enzyme Gene Expression Changes in Young Grass Carp (Ctenopharyngodon idella) Fillets Fed Dietary Valine
Source: PLoS One. 2017 Jan 24;12(1):e0169270. doi: 10.1371/journal.pone.0169270 (PMC5261571; doi:10.1371/journal.pone.0169270)
Supplement: S3 Table — (DOCX) [file pone.0169270.s003.docx]

**S3 Table.** Muscle composition (g kg^-1^) of grass carp supplemented with 4.3, 8.0, 10.6, 13.1, 16.7 and 19.1 g/kg valine (groups 1-6) for 60 days (n=6).

| Number of groups | Ash | | Calcium content | | Phosphorus | |
| --- | --- | --- | --- | --- | --- | --- |
|  | Weight of the sample | Final of the sample weight | Weight of the sample | EDTA(ml) | Weight of the sample | OD |
| 1-1 | 40.2460 | 37.9018 | 2.4974 | 1.35 | 2.4981 | 0.252 |
| 1-2 | 36.5135 | 34.1494 | 2.5189 | 1.25 | 2.4546 | 0.224 |
| 1-3 | 35.6411 | 33.3075 | 2.4832 | 1.45 | 2.5244 | 0.247 |
| 1-4 | 35.4562 | 33.0187 | 2.4114 | 1.50 | 2.5543 | 0.261 |
| 1-5 | 33.4562 | 30.1187 | 2.4714 | 1.45 | 2.4494 | 0.254 |
| 1-6 | 33.0546 | 29.1871 | 2.4611 | 1.55 | 2.4452 | 0.264 |
| 2-1 | 34.5512 | 32.1740 | 2.5166 | 1.35 | 2.4107 | 0.198 |
| 2-2 | 37.1491 | 34.7488 | 2.5366 | 1.40 | 2.5364 | 0.271 |
| 2-3 | 37.6983 | 35.3591 | 2.4900 | 1.15 | 2.4291 | 0.266 |
| 2-4 | 37.8259 | 35.4573 | 2.5045 | 1.25 | 2.4374 | 0.289 |
| 2-5 | 41.3408 | 38.9630 | 2.5289 | 1.34 | 2.5571 | 0.291 |
| 2-6 | 36.3479 | 34.0566 | 2.4498 | 1.10 | 2.515 | 0.276 |
| 3-1 | 38.1534 | 35.8377 | 2.4589 | 1.30 | 2.4513 | 0.264 |
| 3-2 | 38.4226 | 36.0834 | 2.4771 | 1.20 | 2.4358 | 0.263 |
| 3-3 | 43.4710 | 41.0621 | 2.5405 | 1.25 | 2.484 | 0.263 |
| 3-4 | 37.8210 | 35.4567 | 2.5188 | 1.25 | 2.4771 | 0.274 |
| 3-5 | 40.0560 | 37.7287 | 2.4960 | 1.15 | 2.3412 | 0.272 |
| 3-6 | 37.9706 | 35.6020 | 2.5214 | 1.00 | 2.5554 | 0.281 |
| 4-1 | 35.0078 | 32.6198 | 2.5225 | 1.30 | 2.5951 | 0.263 |
| 4-2 | 36.6716 | 34.3202 | 2.5080 | 1.10 | 2.3609 | 0.233 |
| 4-3 | 36.6598 | 34.2717 | 2.5293 | 1.25 | 2.4547 | 0.243 |
| 4-4 | 32.9617 | 30.4197 | 2.6998 | 1.20 | 2.5224 | 0.224 |
| 4-5 | 35.6642 | 33.2898 | 2.5372 | 1.05 | 2.4231 | 0.273 |
| 4-6 | 34.3266 | 30.3290 | 2.4537 | 1.11 | 2.5149 | 0.289 |
| 5-1 | 34.2062 | 31.8075 | 2.5411 | 1.20 | 2.5457 | 0.248 |
| 5-2 | 39.9398 | 37.5631 | 2.5221 | 1.20 | 2.5215 | 0.276 |
| 5-3 | 39.0930 | 36.7133 | 2.5194 | 1.05 | 2.4315 | 0.288 |
| 5-4 | 40.5764 | 38.2807 | 2.4410 | 1.15 | 2.4794 | 0.259 |
| 5-5 | 38.4192 | 36.0447 | 2.5213 | 1.20 | 2.4436 | 0.264 |
| 5-6 | 37.1750 | 34.8066 | 2.5045 | 1.10 | 2.4956 | 0.284 |
| 6-1 | 35.6967 | 33.3283 | 2.5075 | 1.05 | 2.4241 | 0.246 |
| 6-2 | 38.8658 | 36.5041 | 2.5029 | 1.25 | 2.4877 | 0.262 |
| 6-3 | 37.5697 | 35.2207 | 2.4931 | 1.25 | 2.3995 | 0.236 |
| 6-4 | 37.3686 | 34.9415 | 2.5649 | 1.30 | 2.3941 | 0.264 |
| 6-5 | 36.7892 | 34.4174 | 2.5325 | 1.20 | 2.4838 | 0.268 |
| 6-6 | 38.0583 | 35.7147 | 2.5009 | 1.00 | 2.4265 | 0.249 |
