# Supplementary material for: Physical and Flavor Characteristics, Fatty Acid Profile, Antioxidant Status and Nrf2-Dependent Antioxidant Enzyme Gene Expression Changes in Young Grass Carp (Ctenopharyngodon idella) Fillets Fed Dietary Valine
Source: PLoS One. 2017 Jan 24;12(1):e0169270. doi: 10.1371/journal.pone.0169270 (PMC5261571; doi:10.1371/journal.pone.0169270)
Supplement: S4 Table — (DOCX) [file pone.0169270.s004.docx]

**S4 Table.** Malondialdehyde content (MDA, nmol mg^-1^ protein), protein carbonyl content (PC, nmol mg^-1^ protein), activities of superoxide dismutase 1 (SOD1, U mg^-1^ protein), catalase (CAT, U mg^-1^ protein), and reduced glutathione (GSH, mg g^-1^ protein) content of grass carp supplemented with 4.3, 8.0, 10.6, 13.1, 16.7 and 19.1 g/kg valine (groups 1-6) for 60 days (n=6).

| Number of groups | Protein | SOD1 | CAT | GSH | MDA | PC | |
| --- | --- | --- | --- | --- | --- | --- | --- |
|  | estimated value | estimated value | estimated value | estimated value | estimated value | estimated value | control value |
| 1-1 | 0.377 | 0.219 | 1.188 | 0.623 | 0.267 | 0.128 | 0.102 |
| 1-2 | 0.351 | 0.212 | 1.169 | 0.569 | 0.229 | 0.122 | 0.101 |
| 1-3 | 0.356 | 0.250 | 1.200 | 0.554 | 0.245 | 0.126 | 0.101 |
| 1-4 | 0.387 | 0.224 | 1.190 | 0.638 | 0.238 | 0.132 | 0.103 |
| 1-5 | 0.383 | 0.254 | 1.198 | 0.669 | 0.275 | 0.124 | 0.103 |
| 1-6 | 0.395 | 0.215 | 1.189 | 0.696 | 0.260 | 0.128 | 0.102 |
| 2-1 | 0.434 | 0.267 | 1.152 | 0.757 | 0.398 | 0.129 | 0.102 |
| 2-2 | 0.460 | 0.254 | 1.160 | 0.744 | 0.290 | 0.128 | 0.101 |
| 2-3 | 0.432 | 0.245 | 1.132 | 0.784 | 0.284 | 0.123 | 0.102 |
| 2-4 | 0.413 | 0.196 | 1.129 | 0.768 | 0.236 | 0.130 | 0.103 |
| 2-5 | 0.416 | 0.256 | 1.174 | 0.717 | 0.320 | 0.127 | 0.102 |
| 2-6 | 0.410 | 0.257 | 1.161 | 0.690 | 0.268 | 0.126 | 0.102 |
| 3-1 | 0.432 | 0.160 | 1.177 | 0.850 | 0.244 | 0.127 | 0.103 |
| 3-2 | 0.403 | 0.168 | 1.170 | 0.842 | 0.294 | 0.125 | 0.102 |
| 3-3 | 0.430 | 0.227 | 1.162 | 0.864 | 0.277 | 0.127 | 0.102 |
| 3-4 | 0.423 | 0.219 | 1.151 | 0.844 | 0.358 | 0.128 | 0.102 |
| 3-5 | 0.451 | 0.210 | 1.175 | 0.863 | 0.308 | 0.125 | 0.103 |
| 3-6 | 0.488 | 0.227 | 1.148 | 0.827 | 0.304 | 0.125 | 0.102 |
| 4-1 | 0.457 | 0.225 | 1.151 | 0.868 | 0.250 | 0.117 | 0.102 |
| 4-2 | 0.471 | 0.217 | 1.133 | 0.871 | 0.257 | 0.117 | 0.102 |
| 4-3 | 0.446 | 0.243 | 1.148 | 0.880 | 0.260 | 0.115 | 0.102 |
| 4-4 | 0.438 | 0.275 | 1.152 | 0.817 | 0.295 | 0.115 | 0.102 |
| 4-5 | 0.516 | 0.232 | 1.136 | 0.887 | 0.406 | 0.116 | 0.102 |
| 4-6 | 0.468 | 0.216 | 1.129 | 0.811 | 0.260 | 0.118 | 0.102 |
| 5-1 | 0.445 | 0.206 | 1.168 | 0.782 | 0.238 | 0.119 | 0.102 |
| 5-2 | 0.450 | 0.226 | 1.190 | 0.686 | 0.268 | 0.121 | 0.103 |
| 5-3 | 0.463 | 0.249 | 1.189 | 0.721 | 0.292 | 0.117 | 0.103 |
| 5-4 | 0.411 | 0.263 | 1.177 | 0.707 | 0.299 | 0.118 | 0.103 |
| 5-5 | 0.417 | 0.254 | 1.184 | 0.697 | 0.278 | 0.118 | 0.103 |
| 5-6 | 0.417 | 0.213 | 1.166 | 0.700 | 0.270 | 0.119 | 0.102 |
| 6-1 | 0.401 | 0.215 | 1.175 | 0.632 | 0.219 | 0.118 | 0.101 |
| 6-2 | 0.435 | 0.262 | 1.170 | 0.655 | 0.244 | 0.118 | 0.102 |
| 6-3 | 0.424 | 0.237 | 1.176 | 0.676 | 0.261 | 0.120 | 0.102 |
| 6-4 | 0.421 | 0.253 | 1.156 | 0.689 | 0.245 | 0.122 | 0.102 |
| 6-5 | 0.424 | 0.267 | 1.187 | 0.665 | 0.233 | 0.120 | 0.102 |
| 6-6 | 0.396 | 0.242 | 1.154 | 0.600 | 0.260 | 0.121 | 0.102 |
