# Supplementary material for: Physical and Flavor Characteristics, Fatty Acid Profile, Antioxidant Status and Nrf2-Dependent Antioxidant Enzyme Gene Expression Changes in Young Grass Carp (Ctenopharyngodon idella) Fillets Fed Dietary Valine
Source: PLoS One. 2017 Jan 24;12(1):e0169270. doi: 10.1371/journal.pone.0169270 (PMC5261571; doi:10.1371/journal.pone.0169270)
Supplement: S5 Table — (DOCX) [file pone.0169270.s005.docx]

**S5 Table.** Copper/zinc superoxide dismutase (SOD1), catalase (CAT) and Selenium-dependent glutathione peroxydase (Se-GPx), NF-E2-related factor 2(Nrf2) and Kelch-like- ECH-associated protein 1 (keap1), target of rapamycin (TOR) and S6 Kinase 1(S6K1) CT value of grass carp supplemented with 4.3, 8.0, 10.6, 13.1, 16.7 and 19.1 g/kg valine (groups 1-6) for 60 days (n=6).

| Number of groups | SOD1 | CAT | Se-GPx | Nrf2 | Keap1 | TOR | S6K1 |
| --- | --- | --- | --- | --- | --- | --- | --- |
|  | Ct value | Ct value | Ct value | Ct value | Ct value | Ct value | Ct value |
| 1-1 | 27.01 | 30.32 | 21.80 | 33.38 | 27.81 | 26.19 | 25.95 |
| 1-2 | 26.97 | 30.27 | 21.63 | 33.19 | 27.45 | 26.22 | 25.84 |
| 1-3 | 25.14 | 29.22 | 21.03 | 32.50 | 27.42 | 25.37 | 25.97 |
| 1-4 | 24.96 | 29.09 | 21.21 | 33.85 | 27.64 | 26.21 | 26.25 |
| 1-5 | 26.84 | 29.23 | 22.13 | 32.55 | 27.22 | 25.70 | 26.04 |
| 1-6 | 27.84 | 29.93 | 21.33 | 32.97 | 27.82 | 26.17 | 25.94 |
| 2-1 | 26.72 | 29.06 | 21.34 | 32.18 | 28.05 | 26.65 | 26.15 |
| 2-2 | 26.81 | 29.19 | 21.83 | 31.28 | 27.63 | 25.71 | 26.26 |
| 2-3 | 27.92 | 27.34 | 20.17 | 32.49 | 28.53 | 26.55 | 26.16 |
| 2-4 | 26.82 | 27.21 | 19.92 | 32.99 | 28.55 | 26.20 | 26.10 |
| 2-5 | 25.80 | 28.87 | 22.58 | 32.29 | 28.23 | 26.15 | 26.10 |
| 2-5 | 26.78 | 28.98 | 21.55 | 32.12 | 28.01 | 26.75 | 26.12 |
| 3-1 | 27.08 | 29.71 | 21.08 | 32.07 | 28.72 | 26.01 | 26.07 |
| 3-2 | 27.00 | 29.63 | 20.81 | 31.83 | 27.58 | 25.29 | 26.11 |
| 3-3 | 24.31 | 28.83 | 21.50 | 31.44 | 27.17 | 25.21 | 26.22 |
| 3-4 | 24.68 | 29.08 | 20.97 | 30.24 | 27.77 | 26.70 | 26.06 |
| 3-5 | 27.39 | 28.58 | 21.18 | 31.64 | 27.57 | 24.30 | 26.03 |
| 3-6 | 24.33 | 28.88 | 21.12 | 31.74 | 27.76 | 24.98 | 26.03 |
| 4-1 | 27.63 | 29.24 | 21.98 | 31.94 | 27.60 | 25.20 | 26.07 |
| 4-2 | 27.45 | 29.31 | 20.80 | 32.57 | 28.01 | 25.71 | 26.08 |
| 4-3 | 26.48 | 29.77 | 20.92 | 31.55 | 26.95 | 25.94 | 26.19 |
| 4-4 | 26.65 | 29.65 | 21.00 | 31.64 | 26.89 | 25.09 | 26.16 |
| 4-5 | 27.17 | 29.98 | 21.07 | 32.05 | 26.29 | 25.84 | 26.20 |
| 5-1 | 31.49 | 32.18 | 22.88 | 33.75 | 30.51 | 28.24 | 26.77 |
| 5-2 | 28.97 | 31.98 | 22.90 | 32.31 | 27.62 | 25.40 | 27.72 |
| 5-3 | 26.60 | 30.41 | 21.61 | 31.10 | 26.91 | 25.38 | 26.05 |
| 5-4 | 27.41 | 30.16 | 22.08 | 33.21 | 26.88 | 26.38 | 26.04 |
| 5-5 | 26.30 | 29.29 | 19.95 | 30.91 | 26.23 | 24.38 | 25.80 |
| 5-6 | 27.33 | 29.98 | 22.35 | 31.23 | 28.92 | 27.88 | 26.81 |
| 6-1 | 24.54 | 29.94 | 23.13 | 32.12 | 27.88 | 25.71 | 26.01 |
| 6-2 | 25.61 | 31.53 | 23.03 | 33.70 | 28.09 | 26.07 | 26.09 |
| 6-3 | 24.76 | 31.61 | 22.48 | 32.22 | 27.87 | 26.01 | 26.45 |
| 6-4 | 23.83 | 29.94 | 20.41 | 32.82 | 26.98 | 26.88 | 26.71 |
| 6-5 | 25.81 | 30.18 | 23.24 | 33.02 | 28.08 | 25.82 | 26.55 |
| 6-6 | 24.21 | 29.16 | 23.04 | 32.62 | 27.09 | 26.04 | 26.05 |
